# Typhus Fever

**Published:** 1919-01

**Authors:** 


**Typhus Fever (Observations on a Serological Test : Weil-Felix Reaction).** By C. M. Craig, M.D., and N. Hamilton Fairley, M.D. *The Lancet,* September 21, 1918.

The authors give an account of their experience of the Weil-Felix reaction in a certain number of cases of typhus fever.

*Organism used in this Investigation.* It is a short, Gram-negative, slightly motile, proteus-like bacillus, growing under aerobic conditions only.

Cultures of blood in 12 cases of typhus fever yielded negative results. The urine, feces, and sputum were examined in 21 cases; only once could an organism similar to the bacillus described above be isolated.

*Agglutinating Phenomena in Definite Typhus Fever.* All together, 90 cases were examined; in all but three, agglutination was noted in the first examination. The titres of the agglutinations obtained ranged between 1/10 and 1/2 000, experiments being made from the fourth to the thirteenth day of illness, according to the cases.

*Control Reactions.* The sera of 70 known negative cases were examined for agglutination against this proteus-like organism. They included cases of undulant fever, typhoid, syphilis, relapsing fever, pneumonia and malaria. In 3 cases only, a slight reaction was obtained in a dilution of 1/10.

Tested in a dilution of 1/10 against standard immune serum for B. Shiga, B. Flexner, B. typhosus and paratyphosus A and B, and the cholera vibrio, emulsions of this bacillus gave completely negative results.

*Complement-fixation Reaction.* The development of an agglutinin for this proteus-like organism in the blood of typhus cases is not accompanied by the concomitant development of any immune body, as is demonstrated by the complement-fixation reaction, using as antigen a fresh saline suspension of this organism.

*Animal Experimentation.* Six monkeys were injected with a dose of 1 to 2 cc. of a 24 hours' broth culture of this proteus-like organism. Prior to inoculation all monkeys had negative complement-fixation reactions, and all yielded negative agglutination reactions in dilution of 1/10 against this organism. In all cases a positive complement-fixation reaction was obtained after the injection. Agglutinin developed in the blood serum of the inoculated monkeys together with immune bodies, which had the property of fixing complement in the presence of its specific antigen : namely, the proteus-like organism.

*Significance of Agglutination Reactions in Typhus Cases.* Should this proteus-like organism be necessarily considered as a constant secondary invader in patients infected with typhus fever? This belief is incompatible with the following facts :

(1) Systematic cultural researches with the urine and blood of patients over all stages of the disease do not yield positive results regarding this organism, although it grows very easily on all laboratory media.

(2) Serological investigations of typhus cases failed to demonstrate the presence of any immune body, together with the agglutinin; whereas serological investigations of monkeys inoculated with this organism yield both positive agglutination and complement-fixation reaction.

This agglutination phenomenon may, therefore, be considered as a pseudo-agglutination. Nevertheless, its clinical value is considerable, and this agglutination test must be considered as a most valuable practical aid in the diagnosis of typhus fever.

